# The triangle relationship between human genome, gut microbiome, and COVID-19: opening of a Pandora’s box

**DOI:** 10.3389/fmicb.2023.1190939

**Published:** 2023-06-29

**Authors:** Jie Tong, Yuran Chen, Mei He, Wenjing Wang, Yiyang Wang, Na Li, Qianfeng Xia

**Affiliations:** ^1^Key Laboratory of Tropical Translational Medicine of Ministry of Education, NHC Key Laboratory of Tropical Disease Control, School of Tropical Medicine, Hainan Medical University, Haikou, China; ^2^College of Life Science, Institute of Life Science and Green Development, Hebei University, Baoding, China; ^3^Department of Tropical Diseases, The Second Affiliated Hospital of Hainan Medical University, Haikou, China

**Keywords:** COVID-19, genome, gut microbiota, infectious diseases, SARS-CoV-2

## Abstract

Since the pandemic started, the coronavirus disease 2019 (COVID-19) has spread worldwide. In patients with COVID-19, the gut microbiome (GM) has been supposed to be closely related to the progress of the disease. The gut microbiota composition and human genetic variation are also connected in COVID-19 patients, assuming a triangular relationship between the genome, GM, and COVID-19. Here, we reviewed the recent developments in the study of the relationship between gut microbiota and COVID-19. The keywords “COVID-19,” “microbiome,” and “genome” were used to search the literature in the PubMed database. We first found that the composition of the GM in COVID-19 patients varies according to the severity of the illness. Most obviously, *Candida albicans* abnormally increased while the probiotic Bifidobacterium decreased in severe cases of COVID-19. Interestingly, clinical studies have consistently emphasized that the family Lachnospiraceae plays a critical role in patients with COVID-19. Additionally, we have demonstrated the impact of microbiome-related genes on COVID-19. Specially, we focused on angiotensin-converting enzyme 2’s dual functions in SARS-CoV-2 infection and gut microbiota alternation. In summary, these studies showed that the diversity of GMs is closely connected to COVID-19. A triangular relationship exists between COVID-19, the human genome, and the gut flora, suggesting that human genetic variations may offer a chance for a precise diagnosis of COVID-19, and the important relationships between genetic makeup and microbiome regulation may affect the therapy of COVID-19.

## 1. Introduction

Severe acute respiratory syndrome coronavirus-2 (SARS-CoV-2) has caused significant morbidity, mortality, and socioeconomic disruption worldwide. COVID-19 is an ongoing pandemic that has affected about 572 million confirmed cases and 6 million deaths (WHO, 2021; Xu et al., 2021). The gut microbiome (GM) has been suggested to predict disease severity in patients with COVID-19 (Newsome et al., 2021). Although human gene expression has been linked to the composition of GM, its impact on COVID-19 is largely unknown. Furthermore, the relationship between genes, gut microbes, and COVID-19 remains to be determined.

The risk of acquiring COVID-19 and its adverse effects can be attributed to inherited and acquired variables related to gene expressions. According to a study, the increased prevalence of severe COVID-19 in males may be related to the position of angiotensin-converting enzyme 2 (ACE2) on chromosome X (Gemmati et al., 2020). SARS-CoV down-regulated ACE2 expression in the myocardium can explain myocardial inflammation and injury and adverse cardiac outcome in SARS patients (Oudit et al., 2009). In this context, variants in these genes associated with differences in gene expression and protein function may explain the propensity of an individual to exhibit disease symptoms and the risk of adverse events. Moreover, some researchers hypothesized that regional differences in allele frequencies might explain different incidence and mortality rates (Cao et al., 2020; Yamamoto et al., 2020). These findings identified some specific genes associated with host antiviral immune response and inflammations in COVID-19, both of which may be suitable for targeted therapy with existing drugs.

In addition, the human GM (GM) is closely related to health and disease because of its extensive role in immunity, metabolic transformation, and signaling (Huttenhower et al., 2012; Sharon et al., 2014; Martin et al., 2019; Zheng et al., 2020). Evidence suggests that genetics can influence GM (Blekhman et al., 2015; Kolde et al., 2018). Large-scale human microbial genome-wide association studies have revealed the associations between human GM traits (relative taxon abundance and community diversity) and microbiome-associated variants (Bonder et al., 2016; Wang et al., 2016; Hughes et al., 2020; Kurilshikov et al., 2021; Liu et al., 2021; Rühlemann et al., 2021; Qin et al., 2022). Studies have shown significant changes in the fecal microbiome of patients with COVID-19, characterized by the depletion of beneficial symbionts and enrichment of opportunistic pathogens (Zuo et al., 2020a). Decreased abundance of butyrate-producing bacteria and short-chain fatty acids (SCFA) availability has been associated with severe COVID-19 (Zuo et al., 2021a).

Additionally, an increase of common pathogens in the GM has consistently been related to high infectivity, disease progression, or poor prognosis of COVID-19 patients (Newsome et al., 2021). Studies suggest that the worsening clinical course in patients with COVID-19 infection may partly be due to the activation of severe inflammation by disrupting GM and the growth of pathogenic bacteria (Wang et al., 2021). These results emphasized the importance of managing the GM in patients during and after COVID-19.

Interestingly, as a very important beneficial bacteria in the human gut, the imbalance of the family Lachnospiraceae is closely related to the severity of COVID-19 (Gaibani et al., 2021). Moreover, the study has found that various diseases with a triangular relationship between genes and microbes involve the core gut family Lachnospiraceae that exists in humans from infancy to adulthood (Vacca et al., 2020), which may provide a new perspective for the study of the pathogenesis of the novel coronavirus, as well as the possibility of microbial intervention for the novel coronavirus. Strengthening beneficial species of gut microbiota depleted during COVID-19 may be a new way to mitigate severe disease.

Most samples used for intestinal microflora detection are feces, and the sequencing methods are all shotgun metagenomics sequencing. Not all studies with SARS-CoV-2 infection demonstrate a similar effect on GM. The differences may be due to the composition of the host’s primitive intestinal microflora and the host health status, such as eating patterns, stress levels, and living conditions. While there are still some common features of GM alternations in COVID-19 patients, these will be discussed in the present review. Besides, although animal models (such as mouse models) are commonly used in the GM study related to human disease, fecal samples from SARS-COV-2 patients are more prevalent for gene expression or metabolism studies. Therefore, in the present review, we will focus on the data from SARS-COV-2 patients and not animal models.

Hence, we cautiously present these observations as the hypothesis for this study. We hypothesize that there is a triangle between the genome, GM, and COVID-19. We highlighted important aspects to consider for COVID-19 based on changes in gut microbiota, particularly the intestinal family Lachnospiraceae. We examined gene expression changes in patients during COVID-19 to understand how this knowledge could be applied to the current rapid development of COVID-19 based on genetic mechanisms. Finally, we discussed how to effectively and accurately target COVID-19 therapy based on the relationship between COVID-19 and gene-intestinal flora and briefly commented on possible solutions. The current research will lay the groundwork for paradigm shifts in which clinical studies are needed to assess the COVID-19 pathogenesis and provide important information on reversing COVID-19-induced adverse outcomes by regulating GM composition and gene expression.

## 2. Methods

Our study focused on the original investigation into the relationship between COVID-19 and the human genome and microbiota. The inclusion criterion for this review was data-based research and review articles in which authors used the terms “COVID-19,” “COVID-19 and gut microbiota/gut microbiome,” “COVID-19 and gut genome,” and “COVID-19 and Lachnospiraceae” in their titles or abstracts as well as in the main texts of the publication. Then, 363,197, 672, 120, and 21 articles are identified for the aboving four terms, respectively. All the literature used in this review was collected from the PubMed database. The study concentrated on publications released over the previous 3 years (from Jan 2020 to May 2023).

## 3. Results

### 3.1. The microbiome-related human genetic expression reflects disease severity in COVID-19 patients

The human genetic effect may provide an opportunity to accurately diagnose microbiome-associated disease and highlight genetic background relevance for microbiome modulation and treatment. Nonetheless, further studies need to study whether microbiota profiling can forecast the occurrence of gene-related COVID-19 and whether microbiota regulation can address COVID-19 by regulating gene expression. This review revealed that gastrointestinal inflammation is common in COVID-19 patients. Previous studies have shown that SARS-CoV-2 infected the gastrointestinal tract cells (Carvalho et al., 2020; Jiao et al., 2021; Cheung et al., 2022), suggesting that the mechanism of gastrointestinal injury might be related to the abnormal expression of genes in the gut.

Interestingly, the observation of a recent pilot study showed that *Bacteroides* downregulated the expression of ACE2 in the gut and inversely correlated with SARS-CoV-2 in the fecal samples of patients (Zuo et al., 2020a). The data suggested that *Bacteroides* have the potential protective role against SARS-CoV-2 infection by preventing virus attachment and entry via ACE2. Recent evidence showed that ACE2 is the entry receptor and viral spike initiator protein for SARS-CoV-2 infection, influencing susceptibility and severity of COVID-19 infection (Hoffmann et al., 2020). Angiotensin-converting enzyme 2 (ACE2) was abundantly expressed in intestinal epithelial cells, and SARS-CoV-2 invaded human cell through ACE2 (Penninger et al., 2021). On the other hand, ACE2 plays a crucial role in the negative regulation of gastrointestinal inflammation by controlling the small intestine’s tryptophan absorption (Hashimoto et al., 2012). The study showed that the expression of ACE2, aryl hydrocarbon receptor (AHR), and caspase recruitment domain-containing protein 9 (CARD9) decreased in the gut mucosa, and tryptophan metabolism was impaired in critical COVID-19 patients (Yokoyama et al., 2022). Moreover, gastrointestinal protective microorganisms such as Lactobacillales increased in the critical group (Yokoyama et al., 2022). Paradoxically, the expression of ACE2 in the lung protected mice from lung injury induced by SARS-CoV spike protein by attenuating the renin-angiotensin system (Kuba et al., 2005). ACE2 also inhibited intestinal inflammation in the gut by maintaining amino acids’ homeostasis and intestinal microbiota ecology (Hashimoto et al., 2012), suggesting that ACE2 might play a dual role in the GM mediating susceptibility to COVID-19 infection.

**Figure 1:**
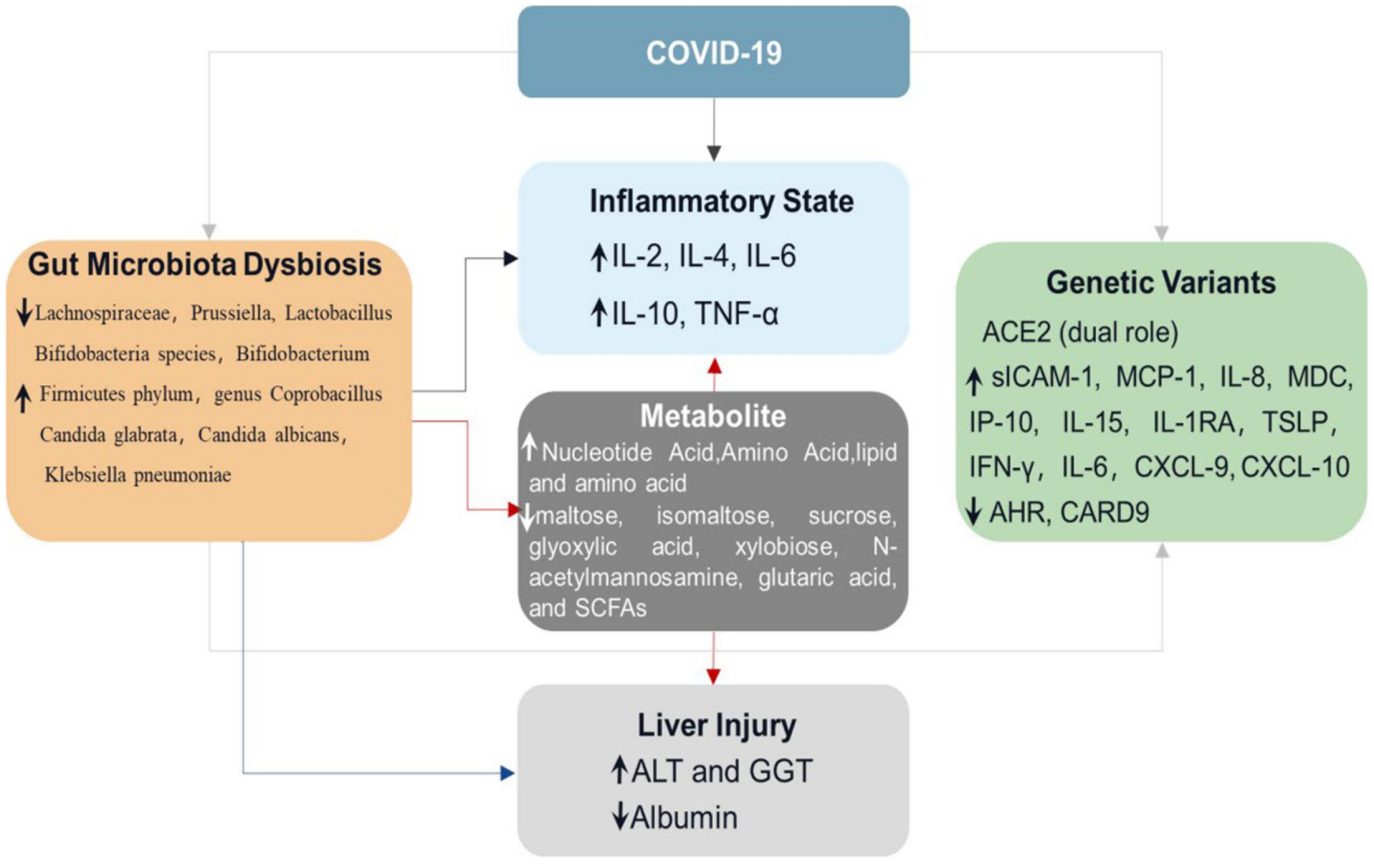
Schematic diagram of diverse intestinal flora in COVID-19 patients. This figure depicts the relationship between COVID-19, intestinal flora, and gene expression. The first line is the activity state of intestinal flora in human health, the second row is the intestinal state after the decrease of the abundance of Spirillum in intestinal flora after infection with COVID-19, and the third row is the change of intestinal flora activity caused by the change of some gene expression in patients with COVID-19. Compared with healthy intestinal microflora, the abundance of Spirillum in patients with COVID-19 decreases, which will not only lead to a significant increase in the level of inflammatory factors in the human body but also increase the level of ALT and GGT, decrease the level of Albumin, and cause certain liver injury. The changes in intestinal flora in patients with COVID-19 will also cause changes in gene expression, resulting in significant changes in the level of inflammatory factors in the blood.

The gut microbiota interacts with the host immune system and metabolic system (Figure 1). Studies have shown that the cytokine levels (sICAM-1, MCP-1, IL-8, IP-10, IL-15, IL-1RA, and TSLP) and metabolites (e.g., quinoline) in the serum increased significantly, and MDC levels of the serum and beneficial microorganisms decreased significantly in the severe COVID-19 patients with fatal outcomes (Albrich et al., 2022). Similarly, there were multiple correlations between the metabolites of gut microbes and COVID-19 (e.g., SCFA, carbohydrates, and neurotransmitters). They were associated with inflammatory cytokines (e.g., IFN-γ, IL-6, and CXCL), which significantly elevated in the severe patients (Nagata et al., 2022). The multi-omics analysis suggested multiple gut microbiota-metabolite-immune function interrelationships in COVID-19 patients (Table 1).

**Table 1:** The effect of microbiome-related human genetic expression on COVID-19.

Demographic features (sex/age)	Severe, no./total (%)	Major respiratory symptom	Organization	Key genetic changes	Effect on microbiota	References
Male sex, no./total (%): 7(47%); median age (years): 55		Fever, Diarrhea cough	Gut	ACE2 was up-regulated	The abundance of Bacteroides decreased	(Zuo et al., 2020a)
Male sex, no./total (%): 13 (68.4); median age (years): 49.6	6 (31.6)	Fever, general fatigue, cough, diarrhea	The small intestinal mucosa	ACE2, AHR, and CARD9 expression decreased in critical COVID-19 patients	The abundance of Lactobacillales increased, and tryptophan metabolism was impaired	(Yokoyama et al., 2022)
Male sex, no./total (%):120(70.0%); median age (years):64.3	130 (75.6)	Nausea/vomiting, diarrhea	Blood	sICAM-1, MCP-1, IL-8, IP-10, IL-15, IL-1RA, and TSLP were up-regulated, and MDC was downregulated in the severe COVID-19 patients with fatal outcomes	The quinolinate and other metabolites increased, and the abundance of Faecalibacterium, Agathobacter, Dorea, Coprococcus, Lachnospiraceae, and Bifidobacterium decreased in the severe COVID-19 patients with fatal outcomes	(Albrich et al., 2022)
Male sex, no./total (%):21(75.0%); median age (years): 52.1	12 (42.9)		Blood	In the severe patients, IFN-γ, IL-6, CXCL-9, and CXCL-10 were significantly elevated	The abundance of *Streptococcus*, *Rothia*, and *Actinomyces* spp. increased, and metabolites included maltose, isomaltose, sucrose, glyoxylic acid, xylobiose, N-acetylmannosamine, glutaric acid, and SCFAs decreased	(Nagata et al., 2022)

### 3.2. GM diversity is a predictor of disease progression in COVID-19 patients

**Figure 2:**
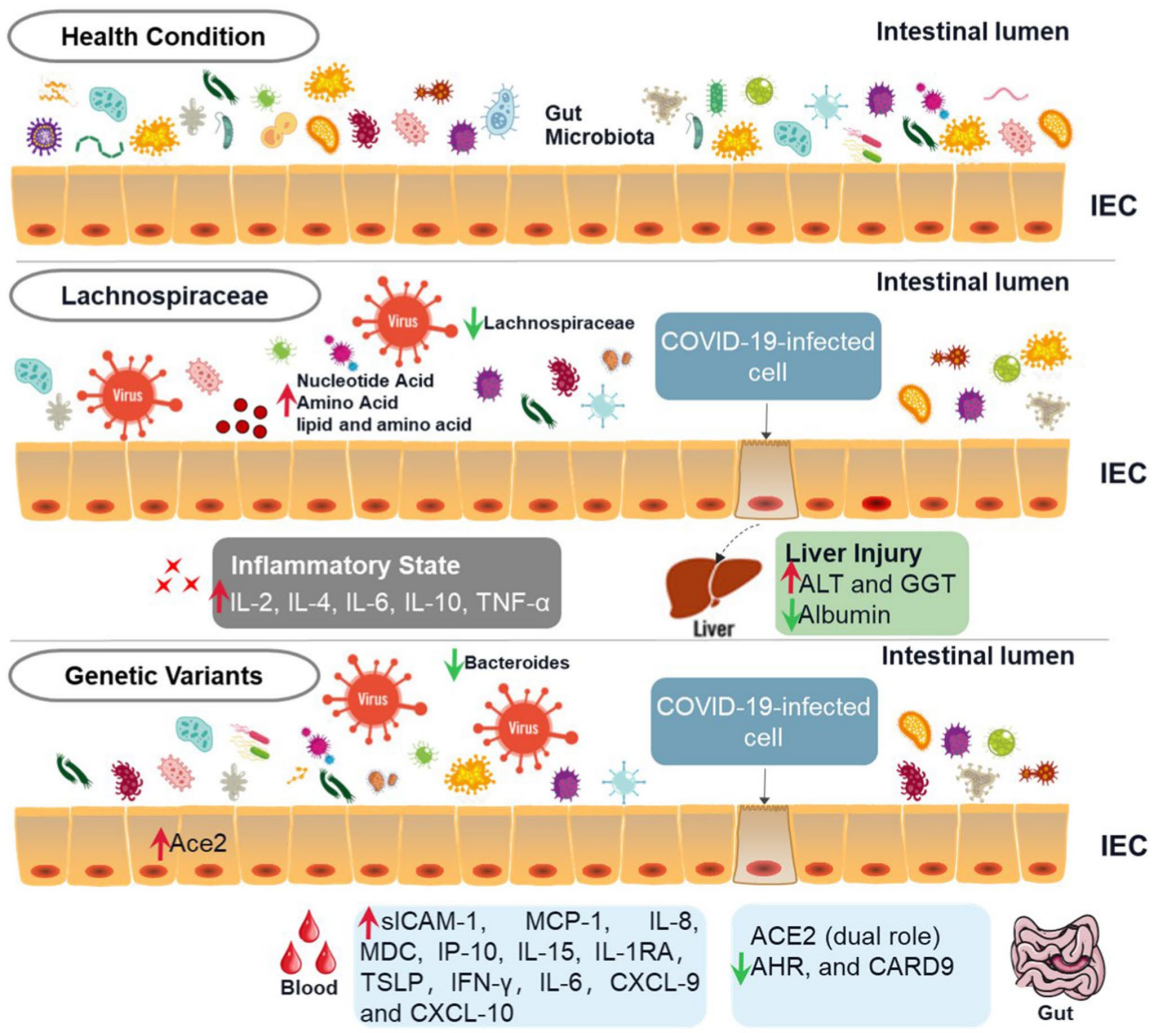
Triangular relationship among COVID-19, GM, and gene expression. When people are infected with SARS-COV-2, the levels of intestinal flora, inflammatory factors, and gene expression will change, and there is a close relationship among them. The changes in intestinal flora in patients with COVID-19 will not only lead to changes in the levels of blood inflammatory factors and metabolites such as nucleotides and amino acids but also lead to changes in gene expression and the levels of ALT, GGT, and other enzymes in the liver, resulting in liver injury. The increased levels of nucleotides, amino acids, and lipoproteins caused by changes in intestinal flora will also lead to increased levels of inflammatory factors and liver injury.

As one of the most important immune organs of humans, the gastrointestinal tract includes a variety of microorganisms, such as bacteria, fungi, and viruses (Zuo et al., 2018; Arbune et al., 2021). Many patients with COVID-19 complain of digestive symptoms like persistent diarrhea (Fandriks, 2010; Xiao et al., 2020). This symptom may be secondary to an inflammatory response caused by the virus (Feng et al., 2020; Hoffmann et al., 2020). As inflammatory cells such as neutrophils and lymphocytes enter the intestinal mucosa, the intestinal microflora may be destroyed, thus causing diarrhea (Xiao et al., 2020). Therefore, the intestinal microflora of humans infected with SARS-CoV-2 is widely changed. As shown in Figure 2, the richness of beneficial symbiotic bacteria and bacteriophages decreased, while the abundance of some harmful microorganisms increased (Xiao et al., 2020; Cao et al., 2021; Zuo et al., 2021b). In COVID-19 patients, the composition of the intestinal microbiome differs, which may relate to the disease severity. Compared with mild to moderate patients, severe to critically ill patients have more opportunistic pathogens (Cao et al., 2021). The severity of COVID-19 disease in COVID-19 patients was positively correlated with the richness of harmful bacteria.

In contrast, the richness of beneficial bacterium was negatively correlated with the severity of the disease (Zuo et al., 2020a, b; Sehli et al., 2021). These discrepancies were independent of the antibiotic treatments, strengthening that GM might be a sufficient predictor of disease severity (Wang et al., 2016; Lai et al., 2020; Sehli et al., 2021). Population-level studies suggested that the inflammatory modulated bacteria was likely to be the signature of disease severity. Specifically, anti-inflammatory bacteria are related to lower severity in virus-infected patients, while pro-inflammatory bacteria might result in higher severity (de Maio et al., 2021).

Among the harmful microorganisms, COVID-19-related candidiasis is a major gut complication (Lai et al., 2020; Salehi et al., 2020; Song et al., 2020; Mastrangelo et al., 2021). According to the study, *Candida albicans* usually cause a superficial skin and mucosal infection, however, in the case of SARS-CoV-2 infection, it will progress to a systemic illness that poses a serious risk of death (Gozalbo et al., 2014; Arastehfar et al., 2020; Lai et al., 2020; Zuo et al., 2020b; Bachtiar et al., 2022). COVID-19 will also damage the human immune response to *Candida albicans*, mainly by weakening the activation of various cellular immune factors (de Maio et al., 2021). On the other hand, the intestinal epithelium damaged by the toxins released by *Candida albicans* will provide a favorable environment for SARS-CoV-2 attachment, which may promote viral replication (Arbune et al., 2021; Aykac et al., 2021). Therefore, *Candida albicans* may be one of the most prevalent gut bacteria in severe COVID-19 patients.

Nevertheless, there was a significant decrease in bacterial diversity and Bifidobacterium abundance in patients with severe COVID-19, which may harm patients’ immune functions. Bifidobacterium is an essential component of probiotic pharmaceuticals. Probiotic therapy can be utilized to treat diarrhea caused by virus infection. By formation of biofilms in the intestines, it can protect the intestinal epithelium from various kinds of toxins (Fitri et al., 2023). Additionally, Bifidobacterium has anti-inflammatory properties that can increase the secretion of anti-inflammatory cytokines and decrease the pro-inflammatory cytokines (Li et al., 2023). Several studies have demonstrated that the presence of Bifidobacterium can aid in the eradication of SARS-COV-2 and lower the mortality rate of COVID-19 patients (d'Ettorre et al., 2020; Ivashkin et al., 2021). Notably, the change in Bifidobacterium abundance may indicate the severity of SARS-CoV-2 infection symptoms (Chen et al., 2020; d'Ettorre et al., 2020; Ivashkin et al., 2021). Some studies have also shown that the presence of Bifidobacterium can assist in the elimination of SARS-CoV-2 pathogens and reduce the mortality of COVID-19 patients (Zuo et al., 2020b, 2021c; Yeoh et al., 2021).

In a word, the infection of SARA-CoV-2 will have a certain impact on the intestinal flora of the human body. Consistent with the alternation of GM, the intestinal microbial ecological network becomes sparse, leading to many SARS-CoV-2 viruses and diverse kinds of opportunistic bacteria, fungi, and eukaryotic viruses enriched in the intestine of patients (Zuo et al., 2021a; Zhang et al., 2022). The dysbiosis of gut microbiota may relate to the severity of COVID-19. Less commonly appreciated is that this phenomenon has existed in the patient for a long, even after the disease has subsided (Aykac et al., 2021). Intestinal microflora also pushes through various mechanisms to affect the spread and progress of the virus infection. Therefore, we can refer to the changes in the species and structure of intestinal microorganisms to infer the disease progression in COVID-19 patients (Table 2). Besides, it enlightens us that during the treatment of COVID-19 patients, we should monitor the abundance of human pathogenic bacteria to prevent bacterial co-infection and provide probiotic adjuvant therapy if necessary.

**Table 2:** Effects of intestinal flora on disease progression in patients with COVID-19.

Disease severity	Demographic features (sex/age)	Outcomes	Changes in intestinal microbiota composition	Biomarker	Different gene expression	References
	Male sex, no./total (%):67 (68%); median age (years): 55.5	Fever 82 (83%) Cough81 (82%) Shortness of breath 31 (31%) Muscle ache 11 (11%) Confusion 9 (9%) Headache 8 (8%) Sore throat 5 (5%) Rhinorrhoea 4 (4%) Chest pain 2 (2%) Diarrhea 2 (2%) Nausea and vomiting 1 (1%) More than one sign or symptom 89 (90%) Fever, cough, and shortness of breath 15 (15%)	Some patients have bacterial and fungal infections, including *Acinetobacter baumannii*, *Klebsiella pneumoniae*, and Aspergillus flavus. Candida glabrata. *Candida albicans*	The concentrations of CRP, Procalcitonin, IL-6, and serum ferritin in the blood were higher than those in normal subjects		(d'Ettorre et al., 2020; Aykac et al., 2021)
47 mild 45 moderate 5 severe 3 critical	Male sex, no./total (%):53 (53%); median age (years):36.4	Fever 38 (38.0%) Diarrhea 17 (17.0%) Cough 40 (40.0%) Sputum 18 (18.0%) Sore throat 8 (8.0%) Rhinorrhea 19 (19.0%) Shortness of breath 9 (9.0%)	The composition of intestinal microorganisms in the patients changed significantly. A variety of intestinal symbiotic bacteria with known immunomodulatory potential, such as Bifidobacterium, were insufficient in the patients and were still very low after the disease subsided	The blood concentrations of CRP, GGT, AST, CXCL10, IL1, TNF-α, and LDH were higher than those in normal subjects		(Zuo et al., 2021c)
	Male sex, no./total (%):17 (57%); median age (years): 55	Fever 26 (86.7%) Cough 26 (86.7%) Diarrhea 5 (16.7%)	The diversity of bacteria in the intestinal tract decreased significantly, and there were abundant opportunistic bacteria in the feces, such as Streptococcus, Rothia, V eillonella, Erysipelatoclostridium, and Actinomyces. The relative abundance of beneficial symbiotic bacteria decreased significantly, such as Lachnospiraceae and Bifidobacterium	The concentrations of CRP, Procalcitonin, ALT, AST, IL10, IL6, TNF-α, Creatinine, and LDH in the blood were higher than in normal subjects		(al Bataineh et al., 2021)
	Male sex, no./total (%):7 (47%); median age (years): 55	Fever 9 (60%) Cough 11 (73%) Diarrhea 7 (1%) Sputum 5 (33%). Rhinorrhea 3 (20%). Shortness of breath 4 (27%)	The number of pathogenic bacteria increased and enriched, and the number of beneficial symbiotic bacteria decreased, such as Prussiella and Lactobacillus, increased			(Zuo et al., 2020a; Qin et al., 2022)
	Male sex, no./total (%):16 (53%); median age (years): 46	Fever 17 (57%) Cough 20 (67%) Diarrhea 4 (13%) Sputum 9 (30%) Rhinorrhea 5 (17%) Rhinorrhea 5 (17%) Shortness of breath 4 (13%)	*Candida albicans* enrichment and highly heterogeneous flora structure occurred in the intestinal microflora of some patients			(Sehli et al., 2021)
	Male sex, no./total (%):29 (48%); median age (years): 42.6	Fever 35 (53%) Diarrhea 8 (12%) Cough 24 (36.4%) Sputum 12 (18.2%) Rhinorrhea (runny nose) 14 (21.2%)	The patients with COVID-19 were primarily characterized by a depletion of *Bifidobacterium adolescentis*, *Ruminococcus bromii*, and F prausnitzii and enrichment of *Bacteroides ovatus*, *Bacteroides dorei*, and *Bacteroides thetaiotaomicron* compared with non–COVID-19 controls	Compared with normal subjects, the ability of intestinal microorganisms to synthesize SCFA and L-isoleucine decreased, and urea synthesis increased in COVID-19 patients		(Yeoh et al., 2021)
3 mild 7 moderate 3 severe	Male sex, no./total (%):6 (46%); median age (years):48		The microbial diversity decreases, the richness of beneficial microorganisms decreases, and the richness of harmful microorganisms increases	The concentrations of C3 and IL-15 in blood were higher than those in normal subjects	PIGR was up-regulated ZEB1 was up-regulated Muc2 was up-regulated TRIB1 was up-regulated	(Zuo et al., 2021b)

### 3.3. The intestinal family Lachnospiraceae is the crucial player in COVID-19 in patients

The gut family Lachnospiraceae exists from infancy to adulthood in humans (Vacca et al., 2020). Lachnospiraceae is enriched in pathway-degrading polysaccharides from dietary sources and is frequently connected with depressive syndromes, inflammatory conditions, and multiple sclerosis (Vacca et al., 2020). Interestingly, as a very important beneficial bacteria in the human gut, the imbalance of the family Lachnospiraceae is closely related to the severity of COVID-19 (Gaibani et al., 2021). Moreover, the study has found that various diseases with a triangular relationship between genes and microbes involve the core gut family Lachnospiraceae that exists in humans from infancy to adulthood (Vacca et al., 2020).

We explore the specific associations between the families of Lachnospiraceae (i. e., Blautia, Bacterium, Roseburia, and Dorea) and COVID-19. The imbalance of Lachnospiraceae is closely related to the severity of COVID-19. Studies indicated that the GM of COVID-19 patients showed a reduction in Lachnospiraceae (Gaibani et al., 2021). In particular, the fecal sample with high SARS-CoV-2 infectivity of SARS-CoV-2 had a lower abundance of Lachnospiraceae than those with low-to-none infectivity (Zuo et al., 2021a). When SARS-CoV-2 infection subsided, the abundance of Lachnospiraceae increased significantly (de Maio et al., 2021; Ren et al., 2021).

This dysbiosis of COVID-19 patients is featured by profound GM destruction with the depletion in the abundance of Lachnospiraceae, which is well known to be related to human health and produces a SCFA (Zhang et al., 2022). Moreover, it is also a microbial metabolite with a key role in human immunological and metabolic homeostasis (Markowitz et al., 2022). Therefore, Lachnospiraceae may contribute to the recovery of patients by reducing the inflammatory cytokine storm and promoting the improvement of the immune system. In contrast, some studies have found that the abundance of Lachnospiraceae was enriched in patients with COVID-19 (al Bataineh et al., 2021; de Maio et al., 2021). Although the results are controversial, Lachnospiraceae certainly plays a very important role in the occurrence and development of COVID-19 (Table 3). More analysis is needed to elucidate the metabolic aspect of the pathophysiology of COVID-19, considering that the microbiota changes of different patients are diverse or even opposite.

**Table 3:** The role of the core intestinal family Lachnospiraceae in the occurrence of COVID-19.

Demographic features (sex/age)	Severe, no./total (%)	Major respiratory symptom	The change of Lachnospiraceae	Genus level	Biomarker	References
Male sex, no./total (%): 38 (55.1%); median age (years): 73		Fever, cough, dyspnoea	A reduction of Lachnospiraceae	Coprococcus, Blautia, Roseburia, and Lachnospira		(Gaibani et al., 2021)
Male sex, no./total (%):7(46.67%); median age (years): 22–75	3(20)	Fever, cough	The abundance of Lachnspiraceae was higher in fecal samples with low-to-none SARS-CoV-2 infectivity than those with high SARS-CoV-2 infectivity	Bacterium	Increased nucleotide and amino acid biosynthesis	(Zuo et al., 2021a)
Male sex, no./total (%): 23 (74%); median age (years): 67		Gastrointestinal symptoms, respiratory failure	Lachnospiraceae was increased after the resolution of SARS-CoV-2 infection	Fusicantibacter and Roseburia		(de Maio et al., 2021)
Male sex, no./total (%): 14 (58%); median age (years): 48		Fever, cough	Lachnospiraceae was increased after the resolution of SARS-CoV-2 infection			(Ren et al., 2021)
Male sex, no./total (%): 52 (61%); age > 18 (100%)			The abundance of *Lachnospiraceae* was enriched in patients with COVID-19	Dorea, Blautia	Enrichment of lipid and amino acid metabolism	(al Bataineh et al., 2021)
Male sex, no./total (%): 24(51%); median age (years): 50			The abundance of *Lachnospiraceae* was enriched in patients with COVID-19	Bacterium		(Li et al., 2021)
Male sex, no./total (%): 43 (46%); median age (years): 52	36 (53.73)	Fever, cough	Lachnospiraceae was negatively correlated with fungi	Agathobacter, Dorea, and Roseburia	Increased IL-2, IL-4, IL-6, IL-10, and TNF-α, lower albumin, higher ALT and GGT	(Lv et al., 2021)

In summary, it has been demonstrated that these genes can shape host-microbe interactions and gut ecology during the pathogenesis of COVID-19. At present, genome-wide association studies on severe COVID-19 have been published (Ellinghaus et al., 2020). However, few studies have shown the impact of microbe-associated human gene expression on COVID-19. We encourage future studies to identify additional SARS-CoV-2 related genes and modulate the dysbiosis induced by SARS-CoV-2 infection for demonstrating reduced lung injury and improved disease prognosis. Further studies on genes and genetic variants are important for preventing and assessing disease severity and individual risk in different populations. The scientific data will provide the basis for developing clinical diagnostic and prognostic tests suitable for high-risk patients with COVID-19. The influence of human genes on microbial abundance and COVID-19 may provide the opportunity for the precise diagnosis and the challenges for the probiotic therapies targetting microbial-related abundance changes.

## 4. Conclusion

In conclusion, with increasing reports indicating GM dysbiosis in COVID-19 patients, the triangle relationship among the GM, human genome, and virus infection will provide novel insights into the clinical management of COVID-19 patients. The disease severity has been reviewed to closely relate to the diversity and abundance of opportunistic gut pathogens. In many severe cases, viral infection provides a favorable environment for the occurrence of bacterial complications, and the existence of opportunistic pathogens will, in turn, promote viral infection, which may finally increase the morbidity and mortality of COVID-19 patients. Detailly, the abundant *Candida albicans* and lessened Bifidobacterium both harm the immune functions of severe COVID-19 patients. Especially the role of Lachnospiraceae should be highlighted. The abundance of Lachnospiraceae will affect the expressions of various host genes like IL-6, IL-4, and TNF-α. The changes in immune-related genes will consequently regulate the antiviral immune response, which may influence the progress of the disease.

Furthermore, ACE2 has been proven to play a dual role in the GM mediating susceptibility to COVID-19 infection. Bacteroides subspecies (Bacteroides subspecies) in fecal microflora of COVID-19 patients weaken the expression of ACE2 in the intestinal tract of mice, which may damage the spike protein-ACE2 interaction to recede virus infection. Another side is the SARS-CoV-2 infection promoting ACE2 modification in the intestine, which increases the susceptibility of the intestinal epithelium to opportunistic pathogens that induce intestinal inflammation and diarrhea. These findings establish triad relationships among the human genome, microbiome, and disease. Consequently, human genetic influences may offer opportunities for precision diagnostics of microbiome-associated diseases but also highlight the relevance of genetic background for microbiome modulation and therapeutics.

## Author contributions

JT, NL, QX, and YC: conceptualization. JT: funding acquisition. YC, MH, WW, YW, and JT: investigation. YC, NL, and JT: writing—original draft. MH, WW, YW, and QX: writing—review and editing. All authors contributed to the article and approved the submitted version.

## Funding

This work was supported by the Central Guidance on Local Science and Technology Development Fund of Hebei Province (226Z2901G), the Hundreds of Talents Program of Hebei Province, China (E2020050011), and the Advanced Talents Incubation Program of the Hebei University (521000981413) to JT.

## Conflict of interest

The authors declare that the research was conducted in the absence of any commercial or financial relationships that could be construed as a potential conflict of interest.

## Publisher’s note

All claims expressed in this article are solely those of the authors and do not necessarily represent those of their affiliated organizations, or those of the publisher, the editors and the reviewers. Any product that may be evaluated in this article, or claim that may be made by its manufacturer, is not guaranteed or endorsed by the publisher.

## Figures and Tables

**Table:** 

COVID-19	Coronavirus disease 2019
SARS-CoV-2	Severe acute respiratory syndrome coronavirus 2
GM	Gut microbiome
ACE2	Angiotensin-converting enzyme 2
AHR	Aryl hydrocarbon receptor
CARD9	Caspase recruitment domain-containing protein 9
GGT	Gamma-glutamyl transferase
SCFA	Short chain fatty acid
AST	Aspartate amino transferase
ALT	Alanine aminotransferase
LDH	Lactate dehydrogenase
CXCL10	C-X-C motif ligand 10
TNF-α	Tumor necrosis factor-α
PIGR	Polymeric immunoglobulin receptor
ZEB1	Zinc finger E-box-binding homeobox 1
TRIB1	Tribbles pseudo kinase 1
IL-1,-6,-10,-15	Interleukin-1,-6,-10,-15
CRP	C-reactive protein
C3	Complement C3
